# Identification and testing of reference genes for Sesame gene expression analysis by quantitative real-time PCR

**DOI:** 10.1007/s00425-012-1805-9

**Published:** 2012-11-16

**Authors:** Libin Wei, Hongmei Miao, Ruihong Zhao, Xiuhua Han, Tide Zhang, Haiyang Zhang

**Affiliations:** Henan Sesame Research Center, Henan Academy of Agricultural Sciences, Zhengzhou, 450002 Henan People’s Republic of China

**Keywords:** BestKeeper, GeNorm, NormFinder, Quantitative real-time PCR, Reference gene, Sesame (*Sesamum indicum* L.)

## Abstract

Sesame (*Sesamum indicum* L.) is an ancient and important oilseed crop. However, few sesame reference genes have been selected for quantitative real-time PCR until now. Screening and validating reference genes is a requisite for gene expression normalization in sesame functional genomics research. In this study, ten candidate reference genes, i.e., *SiACT*, *SiUBQ6*, *SiTUB*, *Si18S rRNA*, *SiEF1α*, *SiCYP*, *SiHistone*, *SiDNAJ*, *SiAPT* and *SiGAPDH,* were chosen and examined systematically in 32 sesame samples. Three qRT-PCR analysis methods, i.e., geNorm, NormFinder and BestKeeper, were evaluated systematically. Results indicated that all ten candidate reference genes could be used as reference genes in sesame. *SiUBQ6* and *SiAPT* were the optimal reference genes for sesame plant development; *SiTUB* was suitable for sesame vegetative tissue development, *SiDNAJ* for pathogen treatment, *SiHistone* for abiotic stress, *SiUBQ6* for bud development and *SiACT* for seed germination. As for hormone treatment and seed development, *SiHistone*, *SiCYP*, *SiDNAJ* or *SiUBQ6*, as well as *SiACT*, *SiDNAJ*, *SiTUB* or *SiAPT,* could be used as reference gene, respectively. To illustrate the suitability of these reference genes, we analyzed the expression variation of three functional sesame genes of *SiSS*, *SiLEA* and *SiGH* in different organs using the optimal qRT-PCR system for the first time. The stability levels of optimal and worst reference genes screened for seed development, anther sterility and plant development were validated in the qRT-PCR normalization. Our results provided a reference gene application guideline for sesame gene expression characterization using qRT-PCR system.

**Electronic supplementary material:**

The online version of this article (doi:10.1007/s00425-012-1805-9) contains supplementary material, which is available to authorized users.

## Introduction

Gene expression analysis is an important and basic step for the systematic understanding of plant biological processes, such as growth and development and biotic and abiotic stress defense pathways. In recent years, quantitative real-time reverse transcriptase PCR (qRT-PCR) has been used as the main analysis technique for quantification and regulating characterization of gene expression. Compared with the traditional method of Northern blot hybridization (Yukawa et al. 1996; Jin et al. 2001; Tai et al. 2002; Chun et al. 2003; Choi et al. 2008; Park et al. 2010), qRT-PCR is an efficient, reliable and sensitive technique for a limited number of target genes (Bustin 2000; Gachon et al. 2004; Hong et al. 2008; Maroufi et al. 2010). To quantify the expression level of a target gene in qRT-PCR, at least one control gene, termed a reference gene, is needed for normalization. Traditional reference genes, such as actin (ACT), ubiquitin 6 (UBQ6), beta-tubulin (TUB), 18S rRNA, elongation factor 1-alpha (EF1α), cyclophilin (CYP), histone, DNAJ-like protein (DNAJ), adenine phosphoribosyl transferase (APT) and glyceraldehyde-3-phosphate dehydrogenase (GAPDH), mostly involving in basic cellular processes, have been widely used as internal controls for gene expression analyses in many crops (Brunner et al. 2004; Nicot et al. 2005; Jain et al. 2006; Jian et al. 2008; Artico et al. 2010; Lee et al. 2010; Qi et al. 2010). While an ideal reference gene would be absolutely valid with a stable expression level in all given tissues and treated conditions (Brunner et al. 2004; Jain et al. 2006), no such universal reference gene has yet been reported. Some studies showed that reference genes did not always keep their stability in any tissues or experimental conditions (Thellin et al. 1999, 2009 Guénin et al. 2009). Selecting several suitable reference genes is necessary for target gene quantification, as the possibility of mismeasures with unvalidated references can be minimized.

Sesame (*Sesamum indicum* L.), which belongs to the *Pedaliaceae* family, is an ancient and important oilseed crop with high oil quality (Chung et al. 2003). Sesame seed is consumed as a traditional health food for its specific antihypertensive effect, hypocholesterolemic activity and antioxidative activity (Coulman et al. 2005; Jan et al. 2009, 2010, 2011; Liao et al. 2009; Mochizuki et al. 2010). However, few reference genes have been selected and validated in sesame until now. During a recent expression survey of a few target genes, the sesame elongation factor gene (EF) was used as the sole reference gene in RT-PCR, even though its preliminary validation had never been performed (Kim et al. 2007, 2010). UBQ5, eIF4A and α-tubulin reported as the optimal reference genes for sesame charcoal rot disease resistance research in March, 2012 have not yet validated with specific sesame genes (Liu et al. 2012). Systematic exploration and validation of more stable sesame reference genes is still requisite.

Therefore, the aims of this study were: (1) to acquire the sequences of ten candidate reference genes based on the new sesame dataset (JP631635–JP668414) or relevant sequence information in other crops, (2) to screen and rank optimal sesame reference genes by their expression variation in 32 different sesame tissues under biotic or abiotic stress conditions and (3) to illustrate the application of the chosen reference genes with three test genes in sesame.

## Materials and methods

### Plant materials

Yuzhi 11 (*Sesamum indicum* L.), a cultivated sesame, was used in the study. Thirty-two different tissues were collected and investigated (Table 1). Vegetative tissue samples including root, stem and leaf were collected at the seedling and flowering stages. Developing buds and seeds were collected through their whole development stages. Callus tissues induced from seed cotyledon and germinating seeds were cultured under proper conditions before being collected. All materials were grown in a greenhouse at 25 °C with 14 h light per day or in the field at the Yuanyang experiment station of Henan Academy of Agricultural Sciences (HAAS).

**Table 1:** Description of 32 samples for qRT-PCR in *Sesamum indicum* L.

Sample no.	Sample type	Growth condition and treatment
S1	Root	Seedling with two pairs of leaves, grown in greenhouse, 25 °C, 14 h light per day
S2	Stem	
S3	Leaf	
S4	Root	Flowering stage, grown in greenhouse, 25 °C, 14 h light per day
S5	Stem	
S6	Leaf	
S7	Root	Seedlings with two pairs of leaves inoculated with 1 × 10^6^ L^−1^ *Fusarium oxysporum* conidiophore suspension for 5 h, grown in greenhouse, 25 °C, 14 h light per day
S8	Stem	
S9	Leaf	
S10	Root	Seedlings with two pairs of leaves, treated with 200 mM NaCl for 5 h, grown in greenhouse, 25 °C, 14 h light per day
S11	Stem	
S12	Leaf	
S13	Root	Seedlings with two pairs of leaves, treated with 20 % PEG 6000 for 5 h, grown in greenhouse, 25 °C, 14 h light per day
S14	Stem	
S15	Leaf	
S16	Root	Seedlings with two pairs of leaves, treated with 4 °C, for 5 h, grown in greenhouse, 25 °C, 14 h light per day
S17	Stem	
S18	Leaf	
S19	Root	Seedlings with two pairs of leaves, treated with 200 μM ABA for 5 h, grown in greenhouse, 25 °C, 14 h light per day
S20	Stem	
S21	Leaf	
S22	Bud, 2 mm	Developing buds with 2–8 mm sizes, grown in experimental field
S23	Bud, 5 mm	
S24	Bud, 8 mm	
S25	5DAF seed	Developing seeds 5–35 days after flowering (DAF), grown in experimental field
S26	15DAF seed	
S27	25DAF seed	
S28	35DAF seed	
S29	Seed germinating 1 day	Seeds were surface-sterilized and cultured on filter paper with distilled water in 1–3 days, 25 °C, 14 h light per day
S30	Seed germinating 2 days	
S31	Seed germinating 3 days	
S32	Callus tissue	Induced from cotyledon and cultured on MS medium with 0.1 mg L^−1^ NAA and 2.0 mg L^−1^ 6-BA and 30 g L^−1^ sucrose

For biotic stress treatment, seedlings with two pairs of leaves were inoculated with 1 ml of ×10^6^ conidiophore suspension of *Fusarium* wilt pathogen (No. HSFO 09030) for 5 h at 25 °C.

For salt and drought stress treatments, seedlings with two pairs of leaves were treated with 200 mM NaCl and 20 % PEG 6000 for 5 h. For cold treatment, the seedlings were cultured at 4 °C for 5 h.

For hormone treatment, seedlings were sprayed with 200 μM abscisic acid (ABA) and were cultured for 5 h.

To verify the expression pattern of the chosen reference genes, ms86-1, a genic male sterile (GMS) line, was cultured in the field and the fertile (plump, white) and sterile (thin, green) anthers were collected under two stages (bud size <4 and >4 mm).

All the collected samples were immersed in liquid nitrogen and stored individually at −70 °C for RNA extraction.

### RNA isolation and cDNA preparation

Total RNA was isolated using the TRIzol reagent (Invitrogen) according to the manufacturer’s instructions. RNA samples were assessed with OD 260/280 > 2.0 and OD 260/230 > 1.8. Equal amounts of total RNA (2 μg) in all samples were treated with gDNA Eraser to eliminate genomic DNA contamination, and then used for cDNA synthesis using a PrimeScript^™^ RT Reagent Kit (Perfect Real Time; TaKaRa). Purified cDNA samples were diluted properly with RNase-free water before used as templates in the qRT-PCR process. RNA extraction and cDNA synthesis from all samples were performed with two biological replicates.

### Selection of sesame candidate reference genes and functional genes

Ten housekeeping sesame genes, including *SiACT*, *SiUBQ6*, *SiTUB*, *Si18S rRNA*, *SiEF1α*, *SiCYP*, *SiHistone*, *SiDNAJ*, *SiAPT* and *SiGAPDH*, were selected as candidate reference genes. Three functional genes of late embryogenesis abundant protein (*SiLEA*), starch synthase (*SiSS*) and glycosyl hydrolase family protein (*SiGH*) were selected for reference gene validation. *Si18S rRNA* sequence was obtained from NCBI data (Accession number: AJ236041). The sequences of other nine reference genes and three validation genes were obtained from our sesame RNA-seq transcriptome dataset (http://www.ncbi.nlm.nih.gov/genbank/TSA.html accession numbers JP631635-JP668414) and compared with the AGI (Arabidopsis Genome Initiative) protein database using BLASTX (http://www.arabidopsis.org/cgi-bin/Blast/TAIRblast.pl; Table 2) with E-value cut-off of 1E−20 as ‘significant matches’.

**Table 2:** Description of ten sesame candidate reference genes for qRT-PCR

Gene name	Gene description	Accession number	*Arabidopsis* homolog locus	E values	Primer and probe sequence (5′–3′)	*T*_m_ (°C)	Amplicon size (bp)	PCR efficiency (%)	Correlation coefficient (*R* ^2^)
*Si18S rRNA*	18S rRNA gene	AJ236041.1	AT3G41768	0	Forward: AGAAACGGCTACCACATCCA	57.9	251	96	0.992
					Reverse: CCAACCCAAGGTCCAACTAC	56.8			
					Probe: *FAM*-AGCAGGCGCGCAAATTACCCAATC-*BHQ1*	69.0			
*SiEF1α*	Elongation factor 1-alpha	JP631636	AT5G60390.3	0	Forward: AAGCCCCTCCGTCTCCCACT	63.0	135	98	0.997
					Reverse: TTCAGTGGTCAAGCCAGATGG	60.0			
					Probe: *FAM*-ATTGGTACTGTCCCCGTTGGTCGTGTG-*BHQ1*	70.0			
*SiACT*	Actin 7	JP631637	AT5G09810.1	E−109	Forward: CTCCCTTTATGCCAGTGGTCGT	61.5	197	102	0.997
					Reverse: GCTCAGCTGTTGTAGTGAAGGA	58.2			
					Probe: *FAM*-CTTGATCTTGCTGGCCGTGATCTCACA-*BHQ1*	69.9			
*SiDNAJ*	DnaJ protein-like	JP631642	AT1G28210.2	2E−70	Forward: CAAAATGGTCCGTTCACACTT	59.9	118	109	0.997
					Reverse: CTGTTTTTGTCCCTTTCACCA	60.0			
					Probe: *FAM*-TACTTGTCCAAATTGCGGAGGAGCTG-*BHQ1*	69.9			
*SiGAPDH*	Glyceraldehyde 3-phosphate dehydrogenase	JP631641	AT3G04120.1	E−155	Forward: GATAAGGCTGCTGCCCACTT	58.0	110	98	0.998
					Reverse: GGCTTGTATTCCTTCTCATTGACA	58.0			
					Probe: *FAM*-CTAAGAAGGTCGTCATCTCTGCCCCGA-*BHQ1*	69.0			
*SiCYP*	Cyclophilin	JP631639	AT3G56070.2	8E−70	Forward: ACAGACCAGGCTCAGTATGCTTT	58.0	103	113	0.997
					Reverse: GGTGGAGACTTCACTAAGGGTAATG	58.0			
					Probe: *FAM*-TTGCTCCATAAATTGATTCTCCCCCAGTC-*BHQ1*	68.0			
*SiTUB*	β-tubulin	JP631640	AT5G23860.2	0	Forward: TGGTGACCTCAACCACCTCAT	59.0	101	105	0.996
					Reverse: TGACAGCGAGTTTCCTGAGATC	58.0			
					Probe: *FAM*-TGTCACATGTTGTCTCCGGTTCCCTG-*BHQ1*	69.0			
*SiAPT*	Adenine phosphoribosyl transferase 1	JP631635	AT1G27450.2	E−76	Forward: TTGCCAATGGACAAAGGGTT	61.5	228	101	0.997
					Reverse: GAGGGTCGGGTCAAGTTAGG	60.9			
					Probe: *FAM*-AGCTGAGCTCACAAGAACAAACAGCG-*BHQ1*	69.0			
*SiUBQ6*	Ubiquitin 6	JP631638	AT2G47110.2	6E−67	Forward: CACCAAGCCGAAGAAGATCAAG	60.0	100	98	0.996
					Reverse: CCTCAGCCTCTGCACCTTTC	59.0			
					Probe: *FAM*-TGAAGCTCGCTGTTCTCCAGTTCTACAAGG-*BHQ1*	69.0			
*SiHistone*	Histone H3	JP631643	AT5G10980.1	3E−41	Forward: CTTGATCAGGAAGTTGCCTTTTC	58.0	101	100	1.000
					Reverse: CCTGAAGCGCCAACACAGCAT	59.0			
					Probe: *FAM*-TTCGCGAAATTGCCCAGGACTTCA-*BHQ1*	69.0			
*SiSS*	Starch synthase	JP631789	AT3G01180.1	E−157	Forward: GGTTGAAGCACAGATGGGTA	58.6	159	105	0.996
					Reverse: CCTGAGAAAGCAAGAGGAGTT	57.8			
					Probe: FAM-TGGATGAGACCACACGGTTCGAATC-BHQ1	69.9			
*SiLEA*	Late embryogenesis abundant protein	JP656886	AT3G15670.1	3E−31	Forward: ATGGCTGATGTGGATGAAGA	59.3	106	102	0.997
					Reverse: AAACAAAGAGCAATACGACCC	58.6			
					Probe: FAM-TGGCCACTAAGAAGATACAGAGGAGATGC-BHQ1	68.4			
*SiGH*	Glycosyl hydrolase family protein	JP647810	AT5G20950.2	0	Forward: TTGAGGAAATGGGTGACGAGA	59.1	185	101	0.990
					Reverse: GGAGGCACAGCAAAAGGGA	59.8			
					Probe: FAM-TGCATGCATCTTCTGTCCGTTCCAA-BHQ1	68.3			

The efficiency and coefficient of determination (*R* ^2^) of qRT-PCR system were determined using LinRegPCR software (*R* ^2^ varied from 0.992 to 1.000 with high efficiency, though the amplicon size of two genes in the qRT-PCR system was larger than 200 bp

### qRT-PCR primer and probe design

qRT-PCR primers for the above thirteen genes were designed using Primer Express 3.0 (ABI) with the melting temperature between 60 and 62 °C and a primer length of 20–26 bp. The length of amplicons ranged from 100 to 251 bp with high polymerization efficiency, which minimized the RNA integrity impact (Fleige and Pfaffl 2006). Meanwhile, specific probes of ten candidate genes with 5′*FAM* and 3′*BHQ1* fluorescence radicals were designed with the melting temperature between 68 and 71 °C, a length of 24–30 bp and about 50 % GC content (Table 2).

### qRT-PCR conditions

To assay the gene expression variability in sesame, qRT-PCR was conducted with an Eppendorf Mastercycler ep Realplex 2.2 Detection System. The PCR reaction volume was 30 μL containing 2.0 μL of diluted cDNA, 0.2 μM of each primer, 0.1 μM probe, 1× PCR buffer, 50 μM of each dNTP and 1.0 U Platinum Taq DNA polymerase (Invitrogen). Reaction mixtures were incubated for 2 min at 37 °C, 5 min at 95 °C, followed by 40 amplification cycles of 15 s at 95 °C and 60 s at 60 °C. All samples were amplified in triplicate times. A negative control without cDNA template was also done at the same time. A standard curve for each gene was generated using tenfold serial dilutions of pooled cDNAs (data not shown). The efficiency of the thirteen pairs of primers in qRT-PCR was calculated using LinRegPCR (Ramakers et al. 2003). To determine their amplicon specificity, electrophoresis analysis of the PCR products was also carried out. Expression levels of the 13 genes in all samples were determined by their cycle threshold values (*C*_t_s).

### Reference gene expression stability determination

Three publicly available software tools, i.e., geNorm (Vandesompele et al. 2002), Normfinder (Andersen et al. 2004) and Bestkeeper (Pfaffl et al. 2004), were used for expression stability determination of the ten genes in sesame.

### GeNorm approach

GeNorm software is a Visual Basic Application (VBA) for ranking the interested genes by the expression stability index, *M*. The least stable gene with the highest *M* value was ranked on the left, and the most stable on the right. The *M* value of a reference gene was not more than 1.5, which is the default limit point (Vandesompele et al. 2002). The pairwise variation (Vn/Vn + 1) between the sequential normalization factors (NF; NFn and NFn + 1) was calculated for determining the optimal number of reference genes (Vandesompele et al. 2002).

### NormFinder approach

Normfinder software, another VBA applet, was used as a model-based approach for identifying the optimal normalization gene(s) among a set of candidates (Andersen et al. 2004). Genes were ranked according to their stability value.

### Bestkeeper approach

BestKeeper, an Excel-based tool, could perform numerous pairwise correlation analyses using raw *C*_t_ values of each gene and estimate inter-gene relations of possible reference gene pairs. The lowest average expression stability value indicated the most stable level (Pfaffl et al. 2004).

## Results

### Identification and characterization of sesame candidate reference genes and functional genes

Compared with the sequences of common reference genes usually used in many plants (Brunner et al. 2004; Nicot et al. 2005; Jain et al. 2006; Jian et al. 2008; Lee et al. 2010; Artico et al. 2010; Qi et al. 2010) and the gene information in GenBank (AJ236041) and our sesame RNA-seq transcriptome data (Zhang et al. 2012), 10 sesame housekeeping genes, including *SiACT*, *SiUBQ6*, *SiTUB*, *Si18S rRNA*, *SiEF1α*, *SiCYP*, *SiHistone*, *SiDNAJ*, *SiAPT* and *SiGAPDH*, were selected as reference gene candidates. The homology levels of ten genes in sesame and *Arabidopsis* were all high with the E values of <1E−20. *SiLEA*, *SiSS* and *SiGH*, which were denominated by *Arabidopsis* homologs, were selected as the validation genes for their specificity in sesame plant development process (Table S1). *SiLEA* and *SiSS* were predominantly expressed in sesame seed and seedling organs, respectively. *SiGH* was mainly expressed in sterile anther rather than fertile anther. Sequences for all of thirteen genes were obtained from GenBank and our sesame RNA-seq dataset (http://www.ncbi.nlm.nih.gov/genbank/TSA.html, accession numbers JP631635- JP668414; Table 2). To ensure their specificity, partial fragments of thirteen genes obtained by RT-PCR were resequenced with the Sanger chain termination method. The results indicated that all amplicons had the same nucleotide sequences as the sequence dataset except for *SiHistone* gene with one nucleotide change (T to G) (data not shown). The qRT-PCR primers and probes of twelve genes were designed by their sequences in the GenBank. As for *SiHistone*, the mutation site was excluded from the primer and probe sequences for qRT- PCR system design (Table 2).

### qRT-PCR specificity and efficiency

To evaluate the amplification specificity and efficiency, electrophoresis was performed for amplicons of the candidate reference genes derived from cDNA and genomic DNA templates in RT-PCR (Fig. S1). All genes generated only single amplicon band from cDNA samples. *SiDNAJ*, *SiACT*, *SiGAPDH* and *SiHistone* of 10 genes showed the amplification differences in amplicon size or number between with genomic DNA templates and cDNA templates, as their primers might span an intron or different gene copies exist in the genome. These four genes could be used for RNA extraction quality assay.

Before carrying out the qRT-PCR, all of the RNA samples with high quality were verified by the PCR results of *SiACT* and *SiGAPDH* genes. There was no genomic DNA contamination in cDNA templates and no amplicons were detected in the negative controls. Neither primer dimers nor unexpected products were found (data not shown). Amplification efficiency ranged from 96 in *Si18S rRNA* to 113 in *SiCYP*, and coefficient of determination (*R* ^2^) varied from 0.992 to 1.000 (Table 2). The qRT-PCR system was demonstrated to be efficient and specific for sesame target gene amplification.

### Expression profiles of ten candidate reference genes

**Fig. 1:**
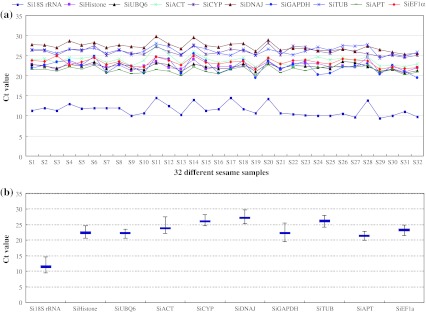
Expression levels of ten candidate reference genes tested in 32 sesame samples. **a** *C*_t_ values of ten candidate reference genes with three replicates. **b** The mean *C*_t_ values of ten candidate reference genes in all sesame samples. The *boxes* represent mean *C*_t_ values. The *bars* indicate the maximum and minimum values

Ten house-keeping genes showed relatively wide ranges of *C*_t_ values, from 11.48 to 27.21 in 32 tested sample pools (Fig. 1), and most *C*_t_ values were between 21.44 and 26.21 (Fig. 1b). The least abundant transcripts were *SiDNAJ* and *SiTUB* with *C*_t_ values of 27.21 and 26.21, respectively. The average *C*_t_ value of the protein encoding genes was approximately 22.64 cycles. Being one of the most abundant RNA species in cell (Guillermo et al. 2011), *Si18S rRNA* expressed highly with its threshold fluorescence point in 11.48 cycles. Also, its transcript level was over 2000-fold higher than the other nine genes. In addition, each candidate gene showed a specific *C*_t_ value variation tendency under the applied conditions. *SiAPT* and *SiUBQ6* showed stable gene expression (below 3 cycles), while *SiGAPDH*, *SiATP* and *Si18S rRNA* had obvious expression variation (above 5 cycles) as shown in Fig. 1b.

### GeNorm analysis

**Fig. 2:**
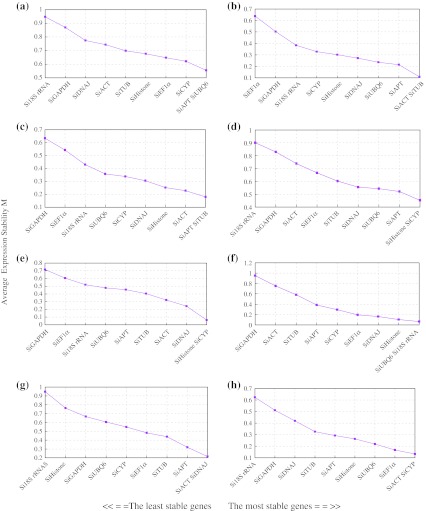
Expression stability values (*M*) of 10 genes in eight sample groups (**a**–**h**) by geNorm software. **a** All sesame samples (S1–S32), **b** vegetative tissues in different developing stages (S1–S6), **c** biotic stress treatment (S7–S9) and normal control (S1–S3), **d** abiotic stress treatment (S10–S18) and normal control (S1–S3), **e** ABA treatment (S19–S21) and normal control (S1–S3), **f** developing buds (S22–S24), **g** developing seeds (S25–S28), **h** germinating seeds (S29–S31)

Before investigating gene expression stability, 32 samples were divided into eight groups by plant development stage and treatment type. We used geNorm software to analyze the expression stability of the tested genes in all samples, and ranked them accordingly to gene stability measure (*M*), which means the genes with the lowest *M* values have the most stable expression (Fig. 2a–h). The result showed that all of the tested genes expressed relatively stably with the *M* value less than 1.0 (Fig. 2a). For all 32 samples, *SiAPT* and *SiUBQ6* showed the lowest *M* value of 0.555, and *Si18S rRNA* was the highest with an *M* value of 0.948. For vegetative tissue development (Fig. 2b), *SiACT* and *SiTUB* genes showed the stability with an *M* value of 0.109; *SiAPT* and *SiTUB* genes ranked the most stable under the sesame *Fusarium* wilt pathogen inoculation with an *M* value of 0.180, while *SiGAPDH* was the least stable with an *M* of 0.634 (Fig. 2c). *SiHistone* and *SiCYP* were the most stable across both abiotic stress (salt, drought and cold) and ABA treatment (Fig. 2d, e). *SiUBQ6* and *Si18S rRNA* were the most stable reference genes with an *M* of 0.065 during bud development (Fig. 2f). As for seed development including seed developing and germinating stages, one of the most stable genes was *SiACT* consistently (Fig. 2g, h).

**Fig. 3:**
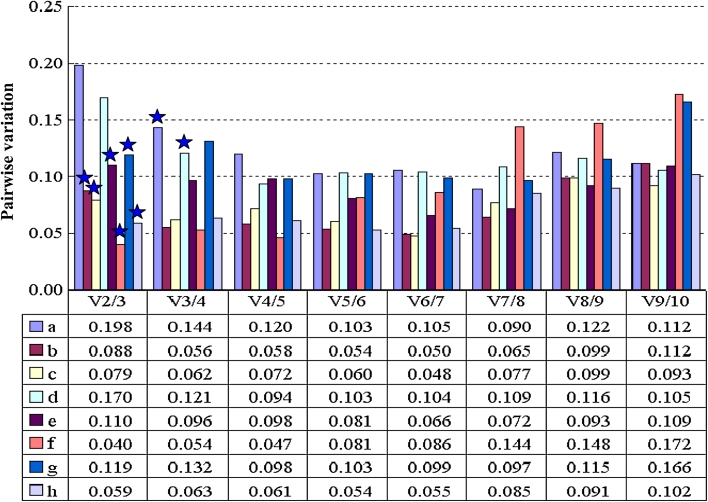
Pairwise variation (*V*) analysis of 10 sesame candidate reference genes in eight sample groups. *Asterisk* indicates the optimal number of reference genes for **a**–**h** sample groups. **a**–**h** Sample groups are the same as in Fig. 2

The pairwise variation, Vn/n + 1, of geNorm software was also used to indicate effects of more new additional reference genes on the PCR normalization. Results showed that only two groups of samples, i.e., the all 32 mixed sample and the abiotic stress treatment sample, showed higher pairwise variation V2/3 value more than 0.15 (Fig. 3). Therefore, three reference genes were necessary and could give more help to gene expression normalization in both group samples.

### NormFinder analysis

To further validate the stability of the reference genes obtained by the geNorm software, we applied the NormFinder software, another VBA approach, for optimal normalization gene(s) identification among the candidates (Andersen et al. 2004). Results indicated that *SiUBQ6* ranked as the most stable gene (stability value = 0.461) and *Si18S rRNA* as the most unstable gene in 32 samples (stability value = 1.097; Table 3), which was consistent with the geNorm analysis. *SiAPT* was optimal with a stability value of 0.115 for the sesame development assay. However, it was noteworthy that *SiDNAJ* showed the same remarkable stability (stability value <0.12) in biotic and abiotic stress treatments, which differed from the results by geNorm. During the seed formation and germination processes, *SiACT* ranked in the top third in NormFinder analysis. In bud development, *SiUBQ6* was considered as the most stable gene. It also meant that different reference genes should be chosen for specific tissues or treatment types.

**Table 3:** Expression stability analysis of ten reference genes in eight sample groups by NormFinder

Rank	a		b		c		d		e		f		g		h	
	Gene name	Stability value	Gene name	Stability value	Gene name	Stability value	Gene name	Stability value	Gene name	Stability value	Gene name	Stability value	Gene name	Stability value	Gene name	Stability value
1	*SiUBQ6*	0.461	*SiAPT*	0.115	*SiDNAJ*	0.119	*SiDNAJ*	0.111	*SiDNAJ*	0.054	*SiUBQ6*	0.057	*SiTUB*	0.126	*EF1α*	0.073
2	*SiEF1α*	0.502	*SiACT*	0.134	*SiEF1α*	0.162	*SiEF1α*	0.159	*SiACT*	0.092	*SiACT*	0.057	*SiDNAJ*	0.145	*SiHistone*	0.097
3	*SiDNAJ*	0.539	*SiUBQ6*	0.169	*SiGAPDH*	0.178	*SiUBQ6*	0.417	*SiEF1α*	0.186	*SiHistone*	0.203	*SiACT*	0.206	*SiACT*	0.111
4	*SiHistone*	0.547	*SiTUB*	0.207	*SiHistone*	0.205	*SiCYP*	0.533	*SiAPT*	0.348	*SiGAPDH*	0.272	*SiEF1α*	0.467	*SiCYP*	0.210
5	*SiCYP*	0.561	*SiDNAJ*	0.255	*Si18S rRNA*	0.226	*SiHistone*	0.612	*SiCYP*	0.488	*SiEF1α*	0.521	*SiAPT*	0.473	*SiUBQ6*	0.217
6	*SiTUB*	0.607	*SiCYP*	0.410	*SiACT*	0.377	*SiAPT*	0.652	*SiHistone*	0.528	*SiDNAJ*	0.737	*SiGAPDH*	0.581	*SiTUB*	0.255
7	*SiACT*	0.619	*SiHistone*	0.427	*SiUBQ6*	0.574	*SiTUB*	0.653	*SiTUB*	0.609	*Si18S rRNA*	0.951	*SiHistone*	0.806	*SiAPT*	0.384
8	*SiAPT*	0.628	*Si18S rRNA*	0.522	*SiCYP*	0.620	*SiACT*	0.889	*Si18S rRNA*	0.611	*SiAPT*	1.031	*SiCYP*	0.839	*SiGAPDH*	0.784
9	*SiGAPDH*	1.054	*SiGAPDH*	0.828	*SiTUB*	0.802	*SiGAPDH*	1.074	*SiUBQ6*	0.624	*SiCYP*	1.295	*SiUBQ6*	0.919	*SiDNAJ*	0.789
10	*Si18S rRNA*	1.097	*SiEF1α*	1.114	*SiAPT*	1.170	*Si18S rRNA*	1.109	*SiGAPDH*	1.329	*SiTUB*	1.811	*Si18S rRNA*	1.645	*Si18S rRNA*	1.010

a–h sample groups were the same as in Fig. 2

### BestKeeper analysis

**Fig. 4:**
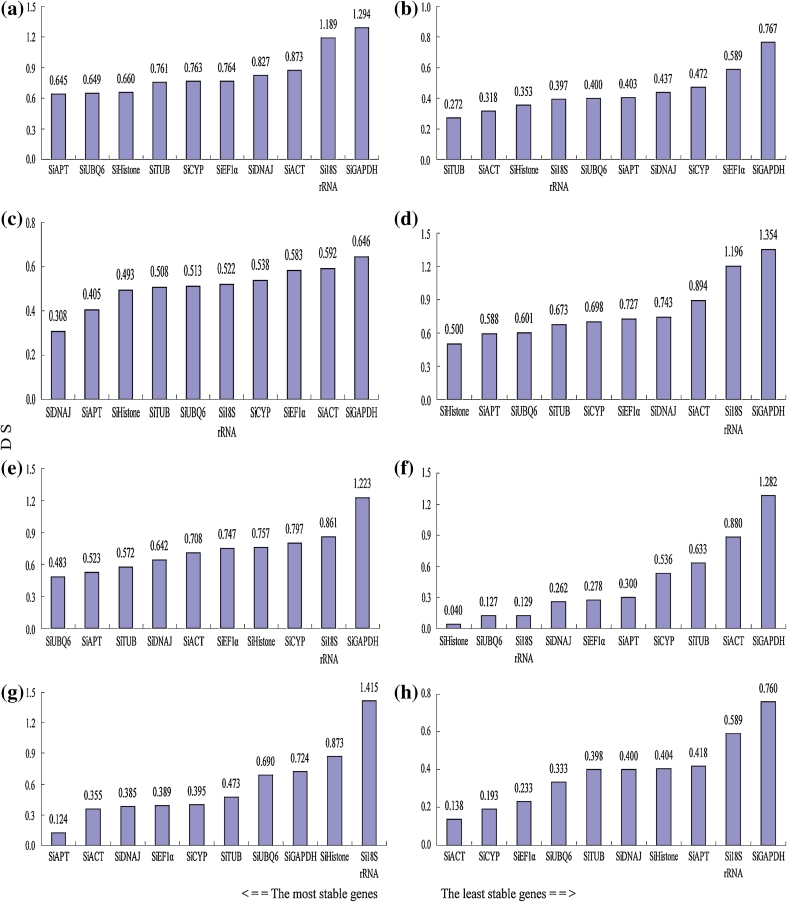
Expression stability analysis of ten reference genes in eight sample groups by BestKeeper. **a**–**h** Sample groups were the same as in Fig. 2. *SD* standard deviation. A lower average expression stability value indicates more stable expression

Being another popular analysis method, BestKeeper was also applied for reference gene expression analysis in this study. Results showed that *SiATP* (SD = 0.645) and *SiUBQ6* (SD = 0.649) were most stable in 32 samples (a), which was almost consistent with the results by geNorm and NormFinder (Fig. 4). For the rest seven group samples (b–h), most stable reference genes were not same as results of above both analysis approaches.

### Testing of sesame reference genes

To illustrate the validation of the above reference genes, as well as investigate the expression levels of sesame functional genes, *SiSS* (JP631789), *SiLEA* (JP656886) and *SiGH* (JP647810) genes with specific temporal or spacial expression characters were chosen from our sesame transcriptome dataset.

**Fig. 5:**
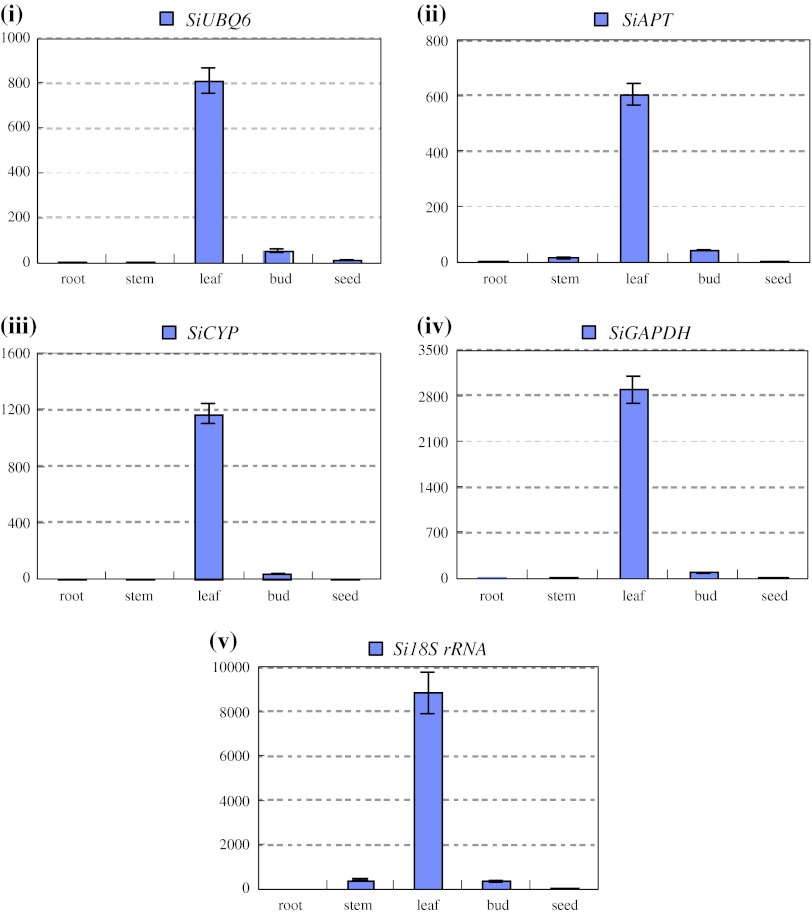
The expression level of the *SiSS* in different plant organs. *SiUBQ6* (**i**), *SiAPT* (**ii**) and *SiCYP* (**iii**) were used as recommended internal controls defined by NormFinder, geNorm and BestKeeper. *SiGAPDH* (**iv**) and *Si18S RNA* (**v**) were used as the worst internal controls accordingly

**Fig. 6:**
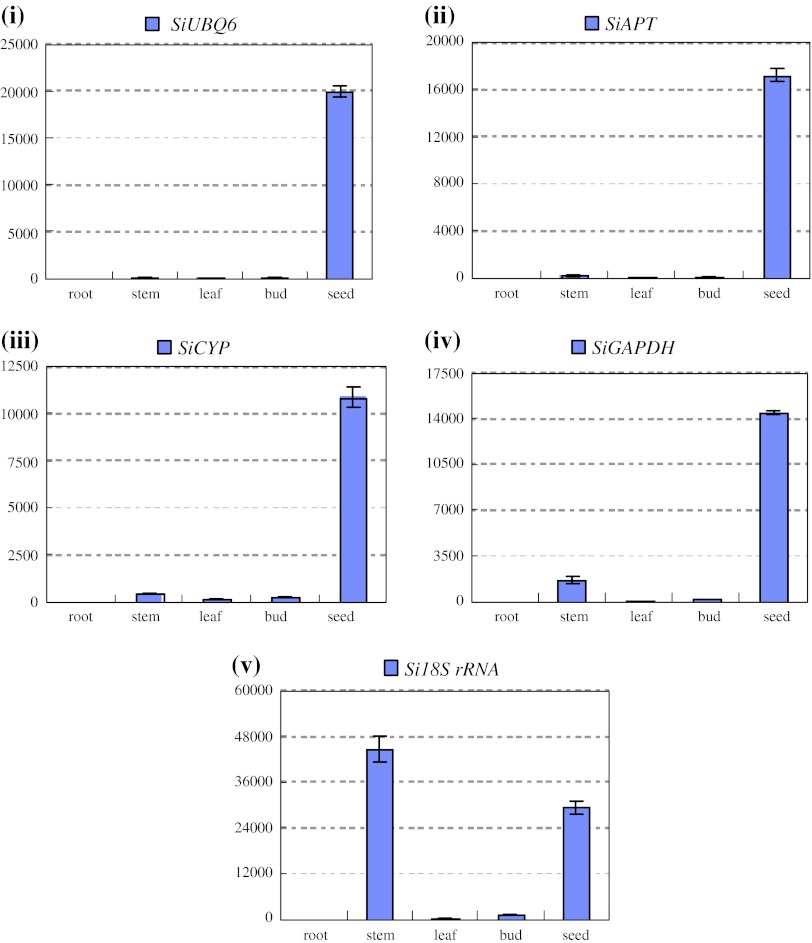
The expression level of the *SiLEA* in different plant organs. *SiUBQ6* (**i**), *SiAPT* (**ii**) and *SiCYP* (**iii**) were used as recommended internal controls defined by NormFinder, geNorm and BestKeeper. *SiGAPDH* (**iv**) and *Si18S RNA* (**v**) were used as the worst internal controls accordingly

**Fig. 7:**
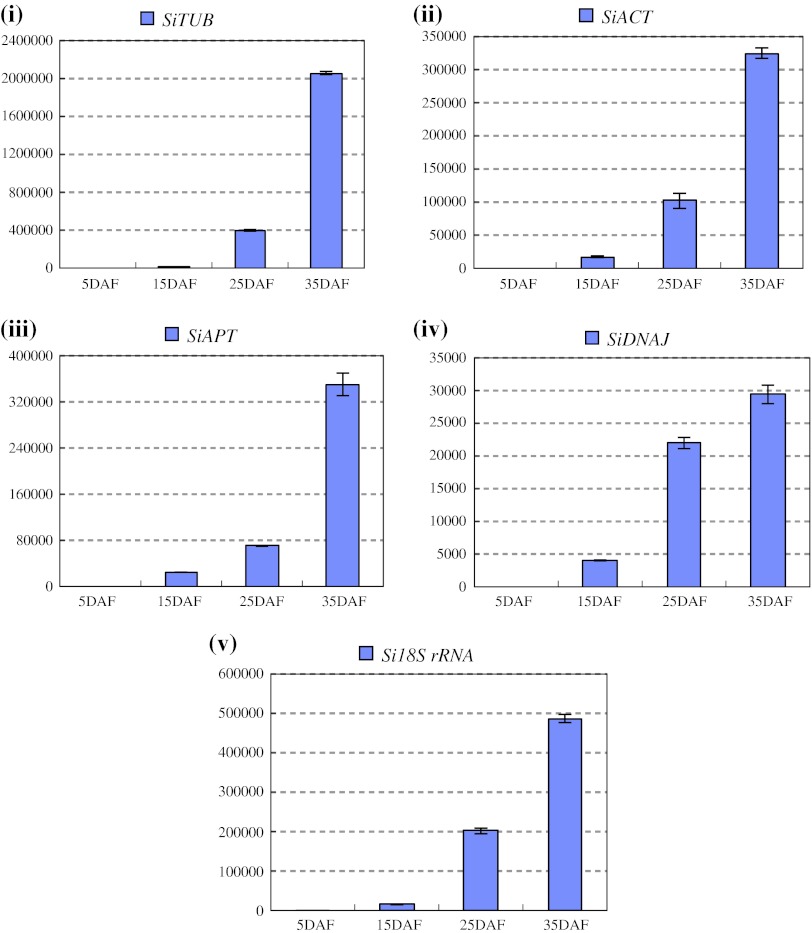
The expression level of the *SiLEA* in developing seed (5–35 DAF) organs. *SiTUB* (**i**), *SiACT* (**ii**), *SiAPT* (**iii**) and *SiDNAJ* (iv) were used as recommended internal controls defined by NormFinder, geNorm and BestKeeper. *Si18S RNA* (**v**) was used as the worst internal controls accordingly

**Fig. 8:**
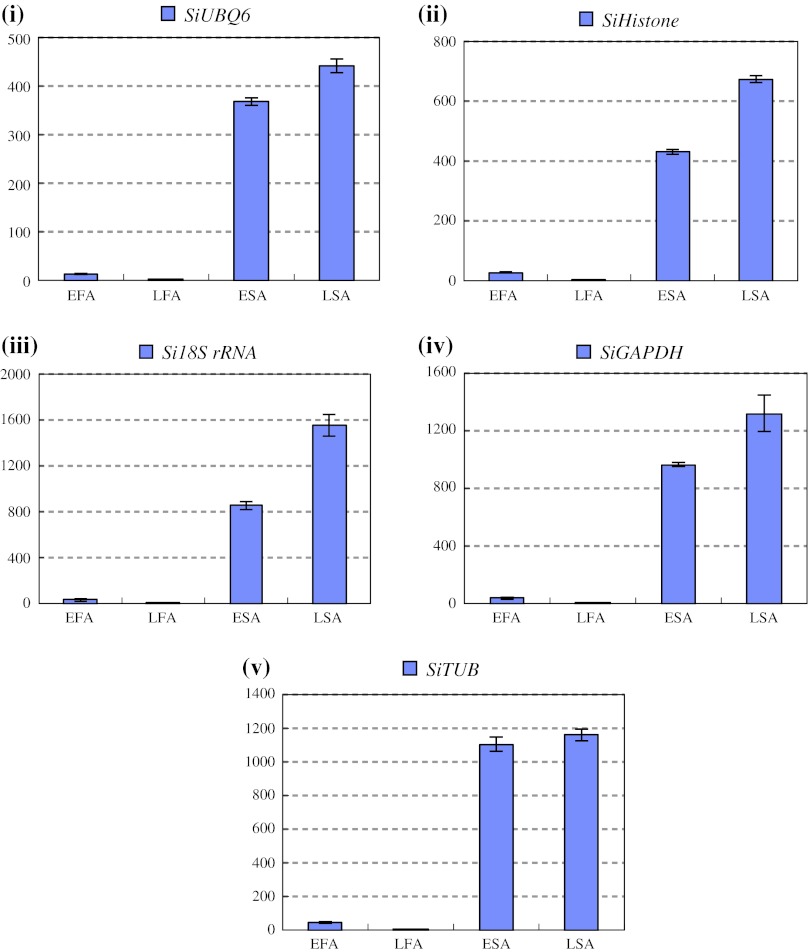
The expression level of the *SiGH* in fertile anthers (*FA*) and sterile anthers (*SA*) of different sizes (early anther with 2.1–4.0 mm length; late anther with 4.1–7.0 mm length). *SiUBQ6* (**i**), *SiHistone* (**ii**) and *Si18S RNA* (**iii**) were used as recommended internal controls defined by NormFinder, geNorm and BestKeeper. *SiGAPDH* (**iv**) and *SiTUB* (**v**) were used as the worst internal controls accordingly

To validate the reliability of the recommended and relative worst reference genes in sesame vegetable growth and development process, we investigated the expression levels of *SiGBSS* and *SiLEA* with *SiAPT*, *SiUBQ6*, *SiCYP* and *Si18S rRNA* for normalizing the qRT-PCR system, respectively (Table 4a). *SiSS* expressed mainly in leaves, rather than in root, stem, bud and seed, with the normalization of either group of the reference genes (Fig. 5). The expression of *SiLEA* normalized with *SiAPT*, *SiUBQ6* and *SiCYP* as reference genes, was predominant in seed; however, the high expression level was also observed in stem except for in seed organ, as *Si18S rRNA*, the worst reference gene, was used for expression normalization (Fig. 6). To further explore the stability of these reference genes, we investigated the expression levels of *SiLEA* during sesame seed development. Four recommended reference genes of *SiTUB*, *SiACT*, *SiAP* and *SiDNAJ*, as well as the worst reference gene of *Si18S rRNA* (Table 4g), were performed in qRT-PCR. The expression patterns of *SiLEA* in four seed- developing stages (5, 15, 25 and 35 DAF) with five different internal controls were consistent, and presented the enhanced tendency in the seed development process (Fig. 7). The results indicated that these recommended references were suitable for sesame-specific gene expression analysis. In some cases, the gene expression pattern could not be normalized with unsuitable reference genes. Furthermore, we analyzed another functional gene, *SiGH*, to validate the reference genes suitable for sesame reproductive growth. *SiGH* was higher in sterile anther compared with fertile anther by the transcriptome dataset (Table S1). Using the recommended reference gene, *SiUBQ6,* *SiHistone* and *Si18S RNA*, as well as the worst reference gene, *SiGAPDH* and *SiTUB* for bud development assay (Table 4f), we compared the expression level of *SiGH* in early and late anther development stages with a sesame nucleic male sterile line, ms86-1 (Fig. 8). The expression variation of *SiGH* gene was just as expected as almost position-specific in sterile anthers rather than fertile anthers. The results further showed that the five reference genes were suitable for sesame gene expression analysis.

**Table 4:** Optimal and worst sesame reference genes in eight sample groups by three methods

Sample groups	Optimal reference gene				Worst reference gene			
	geNorm	Normfinder	BestKeeper	Common gene	geNorm	Normfinder	BestKeeper	Common gene
a	*SiAPT*, *SiUBQ6, SiCYP*	*SiUBQ6*	*SiAPT*	*SiAPT*, *SiUBQ6*	*Si18S rRNA*	*Si18S rRNA*	*SiGAPDH*	*Si18S rRNA*
b	*SiACT*, *SiTUB*	*SiAPT*	*SiTUB*	*SiTUB*	*SiEF1α*	*SiEF1α*	*SiGAPDH*	*SiEF1α*
c	*SiAPT*, *SiTUB*	*SiDNAJ*	*SiDNAJ*	*SiDNAJ*	*SiGAPDH*	*SiAPT*	*SiGAPDH*	*SiGAPDH*
d	*SiHistone*, *SiCYP, SiAPT*	*SiDNAJ*	*SiHistone*	*SiHistone*	*Si18S rRNA*	*Si18S rRNA*	*SiGAPDH*	*Si18S rRNA*
e	*SiHistone*, *SiCYP*	*SiDNAJ*	*SiUBQ6*	None	*SiGAPDH*	*SiGAPDH*	*SiGAPDH*	*SiGAPDH*
f	*SiUBQ6*, *Si18S rRNA*	*SiUBQ6*	*SiHistone*	*SiUBQ6*	*SiGAPDH*	*SiTUB*	*SiGAPDH*	*SiGAPDH*
g	*SiACT*, *SiDNAJ*	*SiTUB*	*SiAPT*	None	*Si18S rRNA*	*Si18S rRNA*	*Si18S rRNA*	*Si18S rRNA*
h	*SiACT*, *SiCYP*	*SiEF1α*	*SiACT*	*SiACT*	*Si18S rRNA*	*Si18S rRNA*	*SiGAPDH*	*Si18S rRNA*

a–h sample groups were the same as in Fig. 2

In conclusion, the optimal sesame reference genes for sesame vegetable and reproductive growth and biotic or abiotic stress conditions were obtained and verified from ten candidate genes. The normalized qRT-PCR system was successfully applicated for the expression pattern analysis of sesame-specific functional genes for the first time.

## Discussion

To investigate sesame reference genes, we selected ten sesame house-keeping genes and observed their relatively wide expression variations from below 3 cycles to more than 5 cycles in this study. Results validated that no single gene among these ten reference genes was ideal for gene transcript normalization in each tissue type and under biotic or abiotic stress conditions. Therefore, more reliable reference gene(s) for various specific conditions are necessary for sesame transcriptome research.

Some reports suggested that applying different analysis software would result in different validation results in the same tissue or treatment, due to their distinct statistical algorithms and analytical procedures (Hong et al. 2008; Hu et al. 2009; Wan et al. 2010). Our results also showed that there was no completely consistent reference gene among the eight (a–h) sample groups using three (geNorm, Normfinder and BestKeeper) analysis approaches in this paper. To analyze gene expression pattern, six common stable genes were chosen and listed as the reference gene(s) for the specific tissues or conditions. *SiUBQ6* and *SiAPT* were the optimal reference genes with the most stable expression level during sesame development. *SiTUB* was suitable for sesame vegetative tissue development analysis, *SiDNAJ* for pathogen treatment, *SiHistone* for abiotic stress, *SiUBQ6* for bud development and *SiACT* for seed germination. As for hormone treatment and seed development, *SiHistone*, *SiCYP*, *SiDNAJ* or *SiUBQ6*, as well as *SiACT*, *SiDNAJ*, *SiTUB* or *SiAPT,* could be used as reference gene, respectively, as no common reliable reference genes were obtained using the three analysis methods.

During testing the suitability of reference genes, *SiSS*, *SiLEA* and *SiGH* genes with specific temporal or spacial expression characters had been used in this study. *SiSS* was annotated by *Arabidopsis* homolog with the E value of E−157. Phylogenetic analysis results showed that *SiSS* was closest with *IbGBSS* (Fig. S2). GBSS contributes to the elongation of glucan chains during starch biosynthesis and has been well characterized in starch crops (Dry et al. 1992; Hylton et al. 1996; Nakamura et al. 1998; Vrinten and Nakamura 2000; Sattler et al. 2009). In cereals, GBSS consists of two isoforms (GBSSI or *waxy* protein and GBSSII) for their different tissue specificity. GBSSI or *waxy* gene expresses exclusively in storage tissues such as seed endosperm and embryo, while the GBSSII gene is transcripted mainly in non-storage tissues such as leaf, stem, root, and pericarp (Dry et al. 1992; Vrinten and Nakamura 2000; Dian et al. 2003). In addition, other different isoforms of GBSS have also been reported in eudicot species (pea, orange, apple and peach), and have various expression profiles differing in cereals (Denyer et al. 1997; Edwards et al. 2002; Szydlowski et al. 2011; Cheng et al. 2012). While validating the normalized qRT-PCR system, we explored and confirmed the tissue specificity of *SiSS* gene in sesame. The *SiSS* gene should belong to GBSS isoform as for its tissue specificity, BLASTX and phylogenetic analysis results.

*SiLEA* gene, which was annotated as the late embryogenesis abundant protein, showed the seed specificity in RNA-seq expression profiles (Table S1) (Zhang et al. 2012). Phylogenetic analysis indicated that *SiLEA* had the high homology with *GmLEA* (Fig. S3). The first LEA gene was characterized in cotton as a set of proteins and highly accumulated in embryos at the maturation phase of seed development (Galau and Dure 1981). To date, many LEA proteins or their genes have been isolated from cotton, barley, wheat, rice and maize (Baker et al. 1988; Curry et al. 1991; Curry and Walker-Simmons 1993; Takahashi et al. 1994; White and Rivin 1995). The gene transcripts are accumulated during late stage seed development and correlated with the seed desiccation stage in other crops (Galau et al. 1987; Hong et al. 1988; Raynal et al. 1989; Hundertmark and Hincha 2008). The seed specificity and expression tendency of *SiLEA* were observed in sesame qRT-PCR (Fig. 7). It also verified the reliability of the recommended reference genes and qRT-PCR system.

Furthermore, we also used *SiGH* gene to validate the qRT-PCR system. Belonging to the glycosyl hydrolases family (Fig. S4), *GH* genes play an important role in many important processes, e.g. fruit ripening, wound and defense responses, pollen maturation and pollen tube growth in plants (Hird et al. 1993; Kang et al. 1994; Duroux et al. 1998; Clément et al. 1999; Moctezuma et al. 2003). Jakobsen et al. (2005) carried out full genome microarray analysis of the wild-type and *mia* mutant anthers transcriptome using Affymetrix chips in *Arabidopsis* and found 17 genes in the glycosyl hydrolase family had strongly increased expression levels in *mia* insertion mutants (Jakobsen et al. 2005). Before this study, we had found that *SiGH* gene expression level probably correlated with sesame male nucleic sterility. In this study, the male sterility expression profile of *SiGH* gene was also revealed in male sterile flower buds in the normalized sesame qRT-PCR system, which was consistent with the expression patterns in cabbage and flax genic male sterile lines (Jungen et al. 2006; Bateer et al. 2009). As a result, the expression profile of *SiSS*, *SiLEA* and *SiGH* functional genes was consistent with results of both our previous RNA-seq and other plants.

In a word, the sesame reference genes obtained in this research were extremely reliable in given tissues and conditions. The gene expression analysis system would provide a guideline for sesame gene function research in the future.

## Electronic supplementary material

Below is the link to the electronic supplementary material.

Supplementary material 1 (DOC 1078 kb)

Supplementary material 2 (DOC 64 kb)

Supplementary material 3 (DOC 68 kb)

Supplementary material 4 (DOC 70 kb)

Supplementary material 5 (DOC 28 kb)

## Abbreviations

ABA: Abscicic acid
ACT: Actin
APT: Adenine phosphoribosyl transferase
Ct: Cycle threshold
CYP: Cyclophilin
DNAJ: DNAJ-like protein
EF1α: Elongation factor 1-alpha
GAPDH: Glyceraldehyde-3-phosphate dehydrogenase
GH: Glycosyl hydrolase family protein
LEA: Late embryogenesis abundant protein
qRT-PCR: Quantitative real-time polymerase chain reaction
R: Coefficient of correlation
SS: Starch synthase
TUB: Beta-tubulin
UBQ6: Ubiquitin 6

## References

[CR1] Andersen CL, Jensen JL, Ørntoft TF (2004). Normalization of real-time quantitative reverse transcription-PCR data: a model-based variance estimation approach to identify genes suited for normalization, applied to bladder and colon cancer data sets. Cancer Res.

[CR2] Artico S, Nardeli SM, Brilhante O, Grossi-de-Sa MF, Alves-Ferreira M (2010). Identification and evaluation of new reference genes in *Gossypium hirsutum* for accurate normalization of real-time quantitative RT-PCR data. BMC Plant Biol.

[CR3] Baker J, dennSteele C, Dure L (1988). Sequence and characterization of 6 Lea proteins and their genes from cotton. Plant Mol Biol.

[CR4] Bateer S, Qiang L, Hui Z, Agula H, Xiaoyun J, Fengyun G (2009). Study on mRNA differential expression in male sterile and fertile flower buds of dominant genomic male sterile flax and sequence analysis of differential fragments. Biotechnol Bull.

[CR5] Brunner AM, Yakovlev IA, Strauss SH (2004). Validating internal controls for quantitative plant gene expression studies. BMC Plant Biol.

[CR6] Bustin SA (2000). Absolute quantification of mRNA using real-time reverse transcription polymerase chain reaction assays. J Mol Endocrinol.

[CR7] Cheng J, Khan MA, Qiu WM, Li J, Zhou H, Zhang Q (2012). Diversification of genes encoding granule-bound starch synthase in monocots and dicots is marked by multiple genome-wide duplication events. PLoS ONE.

[CR8] Choi AM, Lee SB, Cho SH, Hwang I, Hur CG, Suh MC (2008). Isolation and characterization of multiple abundant lipid transfer protein isoforms in developing sesame (*Sesamum indicum* L.) seeds. Plant Physiol Biochem.

[CR9] Chun JA, Jin UH, Lee JW, Yi YB, Hyung NI, Kang MH (2003). Isolation and characterization of a myo-inositol 1-phosphate synthase cDNA from developing sesame (*Sesamum indicum* L.) seeds: functional and differential expression, and salt-induced transcription during germination. Planta.

[CR10] Chung SM, Jung KM, Hur CG, Myung BJ, Park I, Chung CH (2003). Comparative analysis of expressed sequence tags from *Sesamum indicum* and *Arabidopsis thaliana* developing seeds. Plant Mol Biol.

[CR11] Clément C, Pacini E, Audran JC (1999). Anther and pollen: from biology to biotechnology.

[CR12] Coulman KD, Liu Z, Hum WQ, Michaelides J, Thompson LU (2005). Whole sesame seed is as rich a source of mammalian lignan precursors as whole flaxseed. Nutr Cancer.

[CR13] Curry J, Walker-Simmons M (1993). Unusual sequence of group 3 LEA (II) mRNA inducible by dehydration stress in wheat. Plant Mol Biol.

[CR14] Curry J, Morris CF, Walker-Simmons M (1991). Sequence analysis of a cDNA encoding a group 3 LEA mRNA inducible by ABA or dehydration stress in wheat. Plant Mol Biol.

[CR15] Denyer K, Barber L, Edwards E, Smith A, Wang T (1997). Two isoforms of the GBSSI class of granule-bound starch synthase are differentially expressed in the pea plant (*Pisum sativum* L.). Plant Cell Environ.

[CR16] Dian W, Jiang H, Chen Q, Liu F, Wu P (2003). Cloning and characterization of the granule-bound starch synthase II gene in rice: gene expression is regulated by the nitrogen level, sugar and circadian rhythm. Planta.

[CR17] Dry I, Smith A, Edwards A, Bhattacharyya M, Dunn P, Martin C (1992). Characterization of cDNAs encoding two isoforms of granule-bound starch synthase which show differential expression in developing storage organs of pea and potato. Plant J.

[CR18] Duroux L, Delmotte F, Lancelin J, Keravis G, Jay-Allemand C (1998). Insight into naphthoquinone metabolism: β-glucosidase-catalysed hydrolysis of hydrojuglone β-D-glucopyranoside. Biochemical Journal.

[CR19] Edwards A, Vincken JP, Suurs LCJM, Visser RGF, Zeeman S, Smith A (2002). Discrete forms of amylose are synthesized by isoforms of GBSSI in pea. Plant Cell Online.

[CR20] Fleige S, Pfaffl MW (2006). RNA integrity and the effect on the real-time qRT-PCR performance. Mol Asp Med.

[CR21] Gachon C, Mingam A, Charrier B (2004). Real-time PCR: what relevance to plant studies?. J Exp Bot.

[CR22] Galau GA, Dure L (1981). Developmental biochemistry of cottonseed embryogenesis and germination: changing messenger ribonucleic acid populations as shown by reciprocal heterologous complementary deoxyribonucleic acid-messenger ribonucleic acid hybridization. Biochemistry.

[CR23] Galau GA, Bijaisoradat N, Hughes DW (1987). Accumulation kinetics of cotton late embryogenesis-abundant mRNAs and storage protein mRNAs: coordinate regulation during embryogenesis and the role of abscisic acid. Dev Biol.

[CR24] Guénin S, Mauriat M, Pelloux J, Van Wuytswinkel O, Bellini C, Gutierrez L (2009). Normalization of qRT-PCR data: the necessity of adopting a systematic, experimental conditions-specific, validation of references. J Exp Bot.

[CR25] Guillermo M, Mónica S, Vanesa M, Graciela T (2011). Reference gene selection for gene expression studies using RT-qPCR in virus-infected plant hoppers. Virol J.

[CR26] Hird DL, Worrall D, Hodge R, Smartt S, Paul W, Scott R (1993). The anther-specific protein encoded by the *Brassica napus* and *Arabidopsis thaliana* A6 gene displays similarity to β-1,3-glucanases. Plant J.

[CR27] Hong B, Uknes SJ, Ho TD (1988). Cloning and characterization of a cDNA encoding a mRNA rapidly-induced by ABA in barley aleurone layers. Plant Mol Biol.

[CR28] Hong SY, Seo PJ, Yang MS, Xiang F, Park CM (2008). Exploring valid reference genes for gene expression studies in *Brachypodium distachyon* by real-time PCR. BMC Plant Biol.

[CR29] Hu R, Fan C, Li H, Zhang Q, Fu YF (2009). Evaluation of putative reference genes for gene expression normalization in soybean by quantitative real-time RT-PCR. BMC Mol Biol.

[CR30] Hundertmark M, Hincha D (2008). LEA (Late Embryogenesis Abundant) proteins and their encoding genes in *Arabidopsis thaliana*. BMC genomics.

[CR31] Hylton CM, Denyer K, Keeling PL, Chang MT, Smith AM (1996). The effect of waxy mutations on the granule-bound starch synthases of barley and maize endosperms. Planta.

[CR32] Jain M, Nijhawan A, Tyagi AK, Khurana JP (2006). Validation of housekeeping genes as internal control for studying gene expression in rice by quantitative real-time PCR. Biochem Biophys Res Commun.

[CR33] Jakobsen MK, Poulsen LR, Schulz A, Fleurat-Lessard P, Møller A, Husted S (2005). Pollen development and fertilization in *Arabidopsis* is dependent on the MALE GAMETOGENESIS IMPAIRED ANTHERS gene encoding a type V P-type ATPase. Genes Dev.

[CR34] Jan KC, Hwang LS, Ho CT (2009). Biotransformation of sesaminol triglucoside to mammalian lignans by intestinal microbiota. J Agric Food Chem.

[CR35] Jan KC, Ku KL, Chu YH, Hwang LS, Ho CT (2010). Tissue distribution and elimination of estrogenic and anti-Inflammatory catechol metabolites from sesaminol triglucoside in rats. J Agric Food Chem.

[CR36] Jan KC, Ku KL, Chu YH, Hwang LS, Ho CT (2011). Intestinal distribution and excretion of sesaminol and its tetrahydrofuranoid metabolites in rats. J Agric Food Chem.

[CR37] Jian B, Liu B, Bi Y, Hou W, Wu C, Han T (2008). Validation of internal control for gene expression study in soybean by quantitative real-time PCR. BMC Mol Biol.

[CR38] Jin UH, Lee JW, Chung YS, Lee JH, Yi YB, Kim YK (2001). Characterization and temporal expression of a ω-6 fatty acid desaturase cDNA from sesame (*Sesamum indicum* L.) seeds. Plant Sci.

[CR39] Jungen K, Xiaowu W, Guoyu Z, Yanguo Z, Ping L, Zhiyuan F (2006). Sequential expression of fertility-related genes detected by cDNA-AFLP in different types of cabbage male sterile lines. Acta Horticulturae Sinica.

[CR40] Kang IK, Suh SG, Gross KC, Byun JK (1994). N-terminal amino acid sequence of persimmon fruit β-galactosidase. Plant Physiol.

[CR41] Kim MJ, Kim JK, Shin JS, Suh MC (2007). The SebHLH transcription factor mediates trans-activation of the SeFAD2 gene promoter through binding to E-and G-box elements. Plant Mol Biol.

[CR42] Kim MJ, Go YS, Lee SB, Kim YS, Shin JS, Min MK (2010). Seed-expressed casein kinase I acts as a positive regulator of the SeFAD2 promoter via phosphorylation of the SebHLH transcription factor. Plant Mol Biol.

[CR43] Lee JM, Roche JR, Donaghy DJ, Thrush A, Sathish P (2010). Validation of reference genes for quantitative RT-PCR studies of gene expression in perennial ryegrass (*Lolium perenne* L.). BMC Mol Biol.

[CR44] Liao CD, Hung WL, Lu WC, Jan KC, Shih DYC, Yeh AI (2009). Differential tissue distribution of sesaminol triglucoside and its metabolites in rats fed with lignan glycosides from sesame meal with or without Nano/Submicrosizing. Journal of agricultural and food chemistry.

[CR45] Liu LM, Liu HY, Tian BM (2012). Selection of reference genes from sesame infected by *Macrophomina phaseolina*. Acta Agron Sin.

[CR46] Maroufi A, Van Bockstaele E, De Loose M (2010). Validation of reference genes for gene expression analysis in chicory (*Cichorium intybus*) using quantitative real-time PCR. BMC Mol Biol.

[CR47] Mochizuki M, Tsuchie Y, Yamada N, Miyake Y, Osawa T (2010). Effect of sesame lignans on TNF-α-induced expression of adhesion molecules in endothelial cells. Biosci Biotechnol Biochem.

[CR48] Moctezuma E, Smith DL, Gross KC (2003). Antisense suppression of a β-galactosidase gene (TB G6) in tomato increases fruit cracking. J Exp Bot.

[CR49] Nakamura T, Vrinten P, Hayakawa K, Ikeda J (1998). Characterization of a granule-bound starch synthase isoform found in the pericarp of wheat. Plant Physiol.

[CR50] Nicot N, Hausman JF, Hoffmann L, Evers D (2005). Housekeeping gene selection for real-time RT-PCR normalization in potato during biotic and abiotic stress. J Exp Bot.

[CR51] Park MR, Cho E, Rehman S, Yun SJ (2010). Expression of a sesame geranylgeranyl reductase cDNA is induced by light but repressed by abscisic acid and ethylene. Pak J Bot.

[CR52] Pfaffl MW, Tichopad A, Prgomet C, Neuvians TP (2004). Determination of stable housekeeping genes, differentially regulated target genes and sample integrity: BestKeeper–Excel-based tool using pair-wise correlations. Biotechnol Lett.

[CR53] Qi J, Yu S, Zhang F, Shen X, Zhao X, Yu Y, Zhang D (2010). Reference gene selection for real-time quantitative polymerase chain reaction of mRNA transcript levels in Chinese cabbage (*Brassica rapa* L. ssp. pekinensis). Plant Molecular Biology Reporter.

[CR54] Ramakers C, Ruijter JM, Deprez RHL, Moorman AFM (2003). Assumption-free analysis of quantitative real-time polymerase chain reaction (PCR) data. Neurosci Lett.

[CR55] Raynal M, Depigny D, Cooke R, Delseny M (1989). Characterization of a radish nuclear gene expressed during late seed maturation. Plant Physiol.

[CR56] Sattler SE, Singh J, Haas EJ, Guo L, Sarath G, Pedersen JF (2009). Two distinct waxy alleles impact the granule-bound starch synthase in sorghum. Mol Breeding.

[CR57] Szydlowski N, Ragel P, Hennen-Bierwagen TA, Planchot V, Myers AM, Mérida A (2011). Integrated functions among multiple starch synthases determine both amylopectin chain length and branch linkage location in *Arabidopsis* leaf starch. J Exp Bot.

[CR58] Tai SSK, Chen MCM, Peng CC, Tzen JTC (2002). Gene family of oleosin isoforms and their structural stabilization in sesame seed oil bodies. Biosci Biotechnol Biochem.

[CR59] Takahashi R, Joshee N, Kitagawa Y (1994). Induction of chilling resistance by water stress, and cDNA sequence analysis and expression of water stress-regulated genes in rice. Plant Mol Biol.

[CR60] Thellin O, Zorzi W, Lakaye B, De Borman B, Coumans B, Hennen G, Grisar T, Igout A, Heinen E (1999). Housekeeping genes as internal standards: use and limits. J Biotechnol.

[CR61] Thellin O, ElMoualij B, Heinen E, Zorzi W (2009). A decade of improvements in quantification of gene expression and internal standard selection. Biotechnol Adv.

[CR62] Vandesompele J, De Preter K, Pattyn F, Poppe B, Van Roy N, De Paepe A, Speleman F (2002) Accurate normalization of real-time quantitative RT-PCR data by geometric averaging of multiple internal control genes. Genome Biol 3: research0034 10.1186/gb-2002-3-7-research0034 PMC126239 12184808

[CR63] Vrinten PL, Nakamura T (2000). Wheat granule-bound starch synthase I and II are encoded by separate genes that are expressed in different tissues. Plant Physiol.

[CR64] Wan H, Zhao Z, Qian C, Sui Y, Malik AA, Chen J (2010). Selection of appropriate reference genes for gene expression studies by quantitative real-time polymerase chain reaction in cucumber. Anal Biochem.

[CR65] White CN, Rivin CJ (1995). Sequence and regulation of a late embryogenesis abundant group 3 protein of maize. Plant Physiol.

[CR66] Yukawa Y, Takaiwa F, Shoji K, Masuda K, Yamada K (1996). Structure and expression of two seed-specific cDNA clones encoding stearoyl-acyl carrier protein desaturase from sesame, *Sesamum indicum* L. Plant Cell Physiol.

[CR67] Zhang HY, Wei LB, Miao HM, Zhang TD, Wang CY (2012). Development and validation of genic-SSR markers in sesame by RNA-seq. BMC Genomics.

