# Supplementary material for: Identification and testing of reference genes for Sesame gene expression analysis by quantitative real-time PCR
Source: Planta. 2012 Nov 16;237(3):873–89. doi: 10.1007/s00425-012-1805-9 (PMC3579469; doi:10.1007/s00425-012-1805-9)
Supplement: Supplementary file 1 — Supplementary material 1 (DOC 1078 kb) [file 425_2012_1805_MOESM1_ESM.doc]

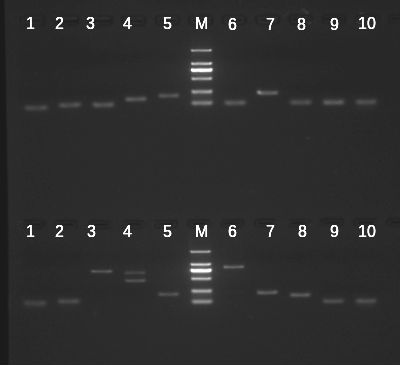


**Fig. S1 Amplification results of ten candidate genes with sesame cDNA and genomic DNA, respectively. Lane**1 *SiCYP*, 2 *SiEF1a*, 3 *SiDNAJ*, 4 *SiACT*, 5 *SiAPT*, 6 *SiGAPDH*, 7 *Si18S rRNA*, 8 *S*i*Histone*, 9 *SiUBQ6*, 10 *SiTUB*. M: DL 2000 marker (from up to low, 2000 bp, 1000 bp, 750 bp, 500 bp, 250 bp, 100 bp). Up lanes indicate the amplification results of ten candidate genes with sesame cDNA as templates. Low lanes indicate the amplification results of ten candidate genes with genomic DNA as templates.
