# Supplementary material for: Identification and testing of reference genes for Sesame gene expression analysis by quantitative real-time PCR
Source: Planta. 2012 Nov 16;237(3):873–89. doi: 10.1007/s00425-012-1805-9 (PMC3579469; doi:10.1007/s00425-012-1805-9)
Supplement: Supplementary file 2 — Supplementary material 2 (DOC 64 kb) [file 425_2012_1805_MOESM2_ESM.doc]

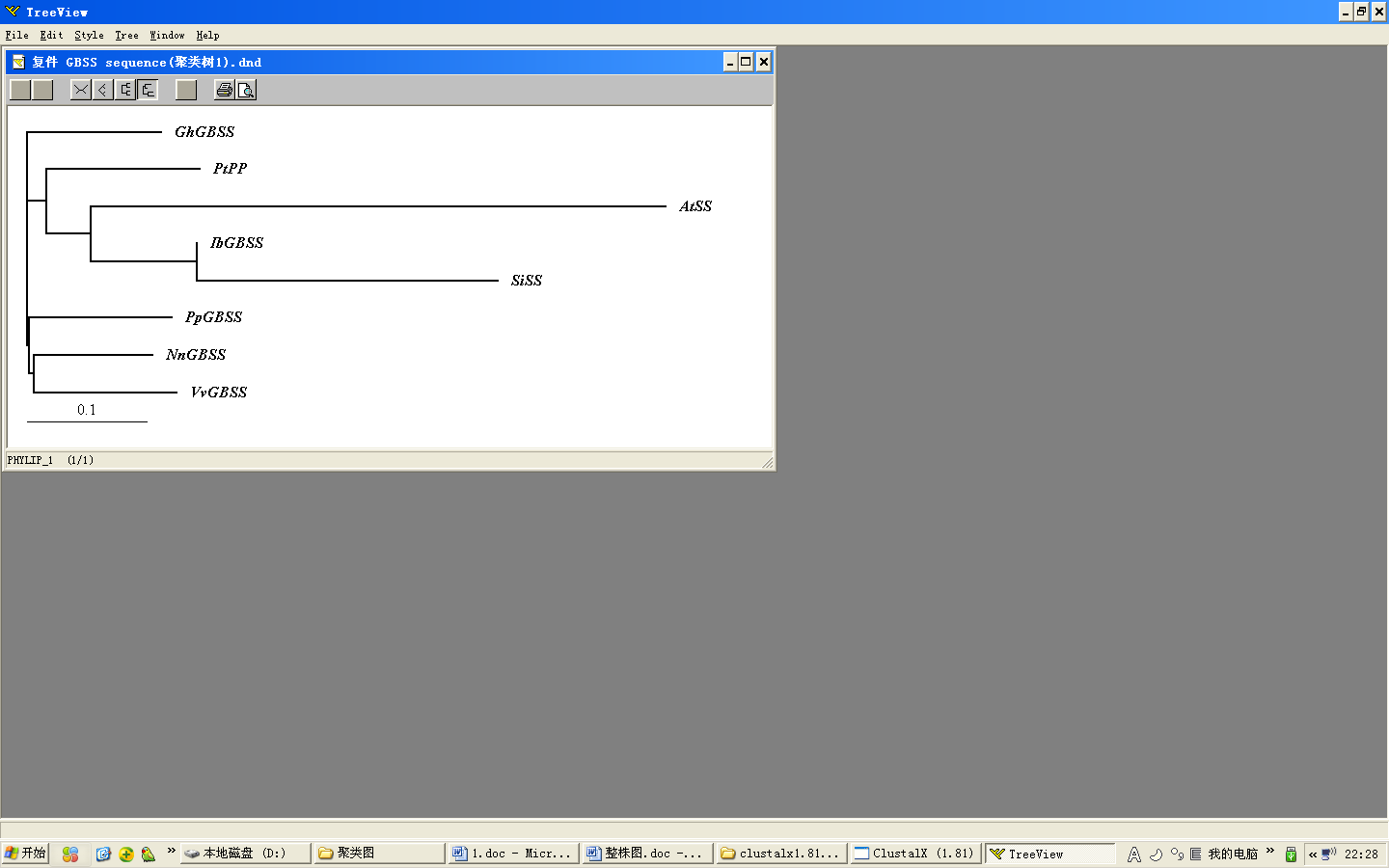


**Fig. S2** **Phylogenetic analysis of *Sesamum indicum* starch synthase gene and other species.** The number below the tree is the value of branch length, which relates to the genetic distance between groups.

Note: *AtSS*: *Arabidopsis thalianas* starch synthase. Accession number: AT3G01180.1; *Ib**GBSS*: *Ipomoea batatas* granule-bound starch synthase. Accession number: BAI83435.1; *NnGBSS*: *Nelumbo nucifera* granule-bound starch synthase. Accession number: ACM78591.1; *PpGBSS*: *Prunus persica* granule-bound starch synthase. Accession number: AFA36447.1; *VvGBSS* :*Vitis vinifera* granule-bound starch synthase. Accession number: XP_003631408.1; *PtPP*: *Populus trichocarpa* predicted protein. Accession number: XP_002300507.1; *GhGBSS*: *Gossypium hirsutum* granule-bound starch synthase. Accession number: ACV72639.1
