# Supplementary material for: Identification and testing of reference genes for Sesame gene expression analysis by quantitative real-time PCR
Source: Planta. 2012 Nov 16;237(3):873–89. doi: 10.1007/s00425-012-1805-9 (PMC3579469; doi:10.1007/s00425-012-1805-9)
Supplement: Supplementary file 3 — Supplementary material 3 (DOC 68 kb) [file 425_2012_1805_MOESM3_ESM.doc]

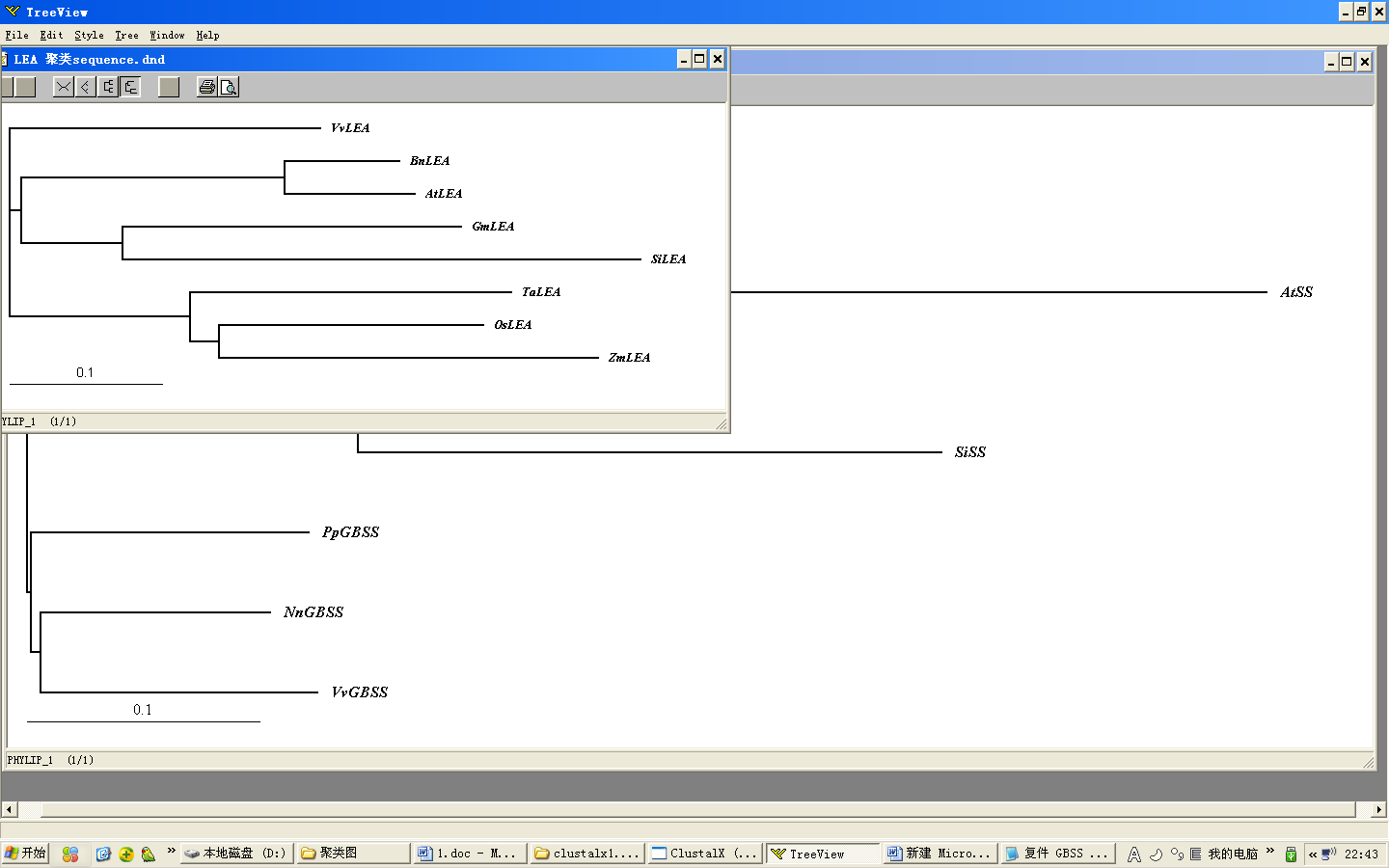


**Fig. S3 Phylogenetic analysis of *Sesamum indicum* late embryogenesis abundant protein gene and other species.** The number below the tree is the value of branch length, which relates to the genetic distance between groups.

Note: *At**LEA*: *Arabidopsis thaliana* late embryogenesis abundant protein. Accession number: AT3G15670.1; *VvLEA*: *Vitis vinifera* late embryogenesis abundant protein*.* Accession number: XP_002285360.1; *BnLEA*: *Brassica napus* late embryogenesis abundant protein*.* Accession number: BAB88877.1; *GmLEA*: *Glycine max* late embryogenesis abundant protein*.* Accession number: XP_003529813.1; *OsLEA*: *Oryza sativa* late embryogenesis abundant protein*.* Accession number: AAD02421.1; *TaLEA*: *Triticum aestivum* late embryogenesis abundant protein*.* Accession number: AAN74637.1; *ZmLEA*: *Zea mays* late embryogenesis abundant protein*.* Accession number: NP_001105298.1
