# Supplementary material for: Identification and testing of reference genes for Sesame gene expression analysis by quantitative real-time PCR
Source: Planta. 2012 Nov 16;237(3):873–89. doi: 10.1007/s00425-012-1805-9 (PMC3579469; doi:10.1007/s00425-012-1805-9)
Supplement: Supplementary file 4 — Supplementary material 4 (DOC 70 kb) [file 425_2012_1805_MOESM4_ESM.doc]

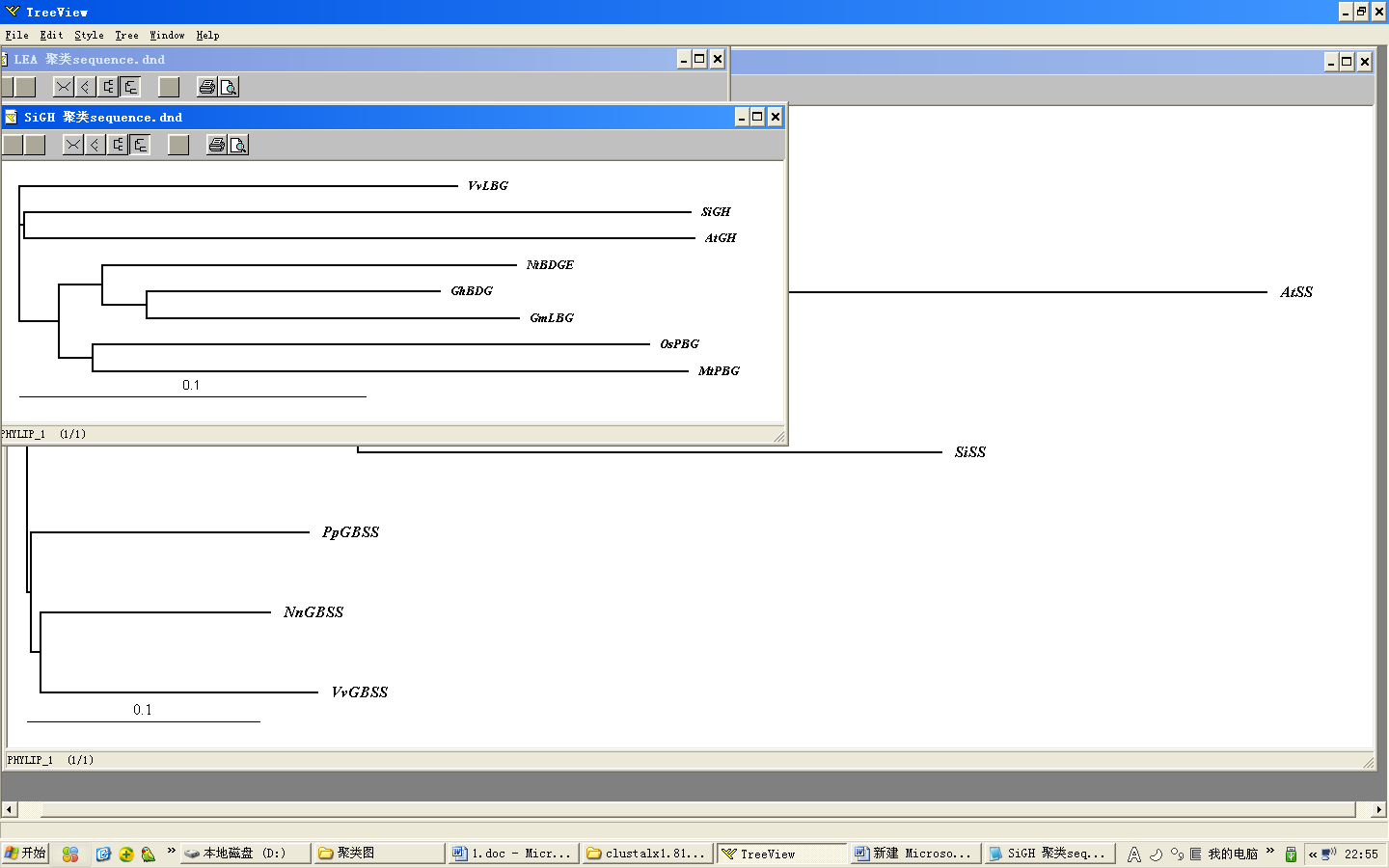


**Fig. S4 Phylogenetic analysis of *Sesamum indicum* glycosyl hydrolases gene and other species.** The number below the tree is the value of branch length, which relates to the genetic distance between groups.

Note: *AtGH*: *Arabidopsis thaliana* glycosyl hydrolase family protein. Accession number: NP_680141.2; *VvLBG*: *Vitis vinifera* lysosomal beta glucosidase. Accession number: XP_002271545.1; *OsPBG*: *Oryza sativa* periplasmic beta-glucosidase. Accession number: NP_001044387.1; *GmLBG*: *Glycine max* lysosomal beta glucosidase. Accession number: XP_003545548.1; *GhBDG*: *Gossypium hirsutum* beta-D-glucosidase. Accession number: AAQ17461.1; *MtPBG*: *Medicago truncatula* periplasmic beta-glucosidase. Accession number: XP_003624652.1; *NtBDGE*: *Nicotiana tabacum* beta-D-glucan exohydrolase. Accession number: BAA33065.1
