# Supplementary material for: Identification and testing of reference genes for Sesame gene expression analysis by quantitative real-time PCR
Source: Planta. 2012 Nov 16;237(3):873–89. doi: 10.1007/s00425-012-1805-9 (PMC3579469; doi:10.1007/s00425-012-1805-9)
Supplement: Supplementary file 5 — Supplementary material 5 (DOC 28 kb) [file 425_2012_1805_MOESM5_ESM.doc]

**Table S1. RNA-seq expression results of three sesame functional genes used in this study**

| **Functional gene name** | **Transcriptome read number in different sesame tissues** | | |
| --- | --- | --- | --- |
|  | Tissue 1 | Tissue 2 | Tissue 3 |
| *SiLEA* | 0 (Seedling) | 1 (Bud) | 5248 (Seed) |
| *SiSS* | 903 (Seedling) | 22 (Bud) | 2 (Seed) |
| *SiGH* | 1 (Fertile anther) | 397 (Sterile anther) | / |

Note: Gene read number indicates how many transcriptome reads can be mapped into the target gene sequence in each specific tissue or sample. All tissues for RNA-seq expression were the same as those in Table 1, except for male sterile line. Fertile and sterile anthers were collected from ms 86-1 line (See the description of “Plant materials” part).
